# The Relationship Between Coronary Collateral Circulation and Serum Adropin Levels

**DOI:** 10.7759/cureus.35166

**Published:** 2023-02-19

**Authors:** Asli Vural, Devrim Kurt, Ahmet Karagöz, Ömer Emecen, Ertan Aydin

**Affiliations:** 1 Department of Cardiology, Giresun University Faculty of Medicine, Giresun, TUR; 2 Department of Cardiology, Samsun University Faculty of Medicine, Samsun, TUR; 3 Department of Biochemistry, Giresun University Faculty of Medicine, Giresun, TUR; 4 Department of Cardiology, Giresun University, Faculty of Medicine, Giresun, TUR

**Keywords:** primary percutaneous coronary intervention (pci), acute non-st elevation myocardial infarction, acute st-elevation myocardial infarction, adropin, coronary collateral circulation

## Abstract

Objective

Coronary collateral circulation (CCC) are vascular structures that limit the infarct area, protect left ventricular function, and reduce the frequency of arrhythmia and mortality during myocardial ischemia and infarction. In this study, we examined the relationship between the development of CCC and serum adropin levels, which has been shown in previous studies to regulate endothelial functions and increase endothelial nitric oxide synthesis, in patients with acute myocardial infarction.

Methods

This study included 41 patients with insufficient CCC and 43 patients with well-developed CCC who were hospitalized for acute myocardial infarction and underwent coronary angiography. The Cohen-Rentrop classification was used to grade the CCC. The patients were divided into two groups according to Rentrop grades: those with a *0-1 stage *were considered as insufficient and those with *grades of 2-3 *were considered as well-developed CCC. We took blood samples to measure the adropin levels within the first 24 hours of hospitalization.

Results

The mean age was 59.1±11.9 years and 62 (73.8%) were male. The right coronary artery was the most frequently target vessel (n: 51, 60.7%), and the majority of the patients presented with ST-segment elevation myocardial infarction (STEMI) (n:58, 69%). The median interval between the severe chest pain and the intervention was significantly higher in patients with well-developed CCC (p=0.042). The serum adropin levels in patients with insufficient CCC were significantly lower than in those with well-developed CCC (196.3 [131.5 - 837.0] pg/mL vs. 235.5 [171.9 - 1124.2] pg/mL, p<0.001). Logistic regression analysis revealed that the circumflex artery as the target vessel, NSTEMI (non-STEMI) as the type of myocardial infarction, and serum adropin level were the independent risk factors for the prediction of poor coronary collateral vessel formation (p<0.05).

Conclusions

In this study, we found that in patients with acute myocardial infarction, those with well-developed CCC had higher adropin levels.

## Introduction

Coronary collateral circulation (CCC) are vessels that are potentially present but are non-functional in a healthy heart. Coronary collaterals have very important and beneficial functions such as reducing ischemia and the frequency of myocardial infarction, limiting the infarct area, preventing aneurysm formation by preserving left ventricular functions, antiarrhythmic effects, and reducing cardiovascular mortality [1-2].

Adropin is a peptide molecule that plays a role in maintaining energy homeostasis and insulin resistance [3]. In recent studies, it has been shown that adropin maintains endothelial function by regulating endothelial nitric oxide synthase and is protective against endothelial dysfunction [4]. An ischemia model was created in animal experiments, and it was observed that adropin treatment increased perfusion and capillary density [5].

There are many studies showing the protective role of adropin on endothelial structure and function. In a recent study, the relationship between adropin level and the development of CCC was shown in patients with chronic coronary syndrome [6]. In this study, we planned to examine the relationship between coronary collateral circulation, which is thought to develop rapidly in patients with acute myocardial infarction, and adropin levels.

## Materials and methods

Methods

This cross-sectional, prospective study included 84 patients, who were admitted to our hospital due to acute myocardial infarction and underwent coronary angiography between January 2019 and December 2020.

Medical treatment was initiated in accordance with the treatment guidelines for patients hospitalized with the diagnosis of acute myocardial infarction (NSTEMI and STEMI) [7-8], and coronary angiography was performed with the conventional method. Patients with acute myocardial infarction with 90% or more stenosis on coronary angiography were included in the study. Those who have previously undergone percutaneous coronary intervention (PCI) or coronary artery bypass grafting; patients with chronic total occlusion, severe kidney and liver disease, pregnancy, malignancy, and chronic inflammatory disease were excluded from the study. Patients with an interval between the last severe chest pain and intervention of more than 12 hours were excluded. This study was approved by the local ethics committee (Clinical Research Ethics Committee of Giresun University) (approval date - number: 04/09/2018 **- **01/07); all patients were informed about the goals of the study and informed consent was obtained.

The presence of CCC towards the artery of the culprit lesion was evaluated in patients with stenosis of 90% or more in coronary angiography and who underwent coronary percutaneous intervention. Angiographic images were evaluated by two experienced cardiologists, and they made a joint decision in the case of borderline lesions.

CCC was graded according to the Rentrop classification: 0: no significant collateral circulation, 1: collateral circulation to the lateral branches without reaching the epicardial artery, 2: partial filling of the epicardial artery, 3: full filling of the epicardial artery [9]. The patients were divided into two groups according to Rentrop grades: those with a Rentrop grade of 2-3were accepted as well-developed collateral circulation, and those with a grade of 0-1 were considered as insufficient collateral circulation. Forty-one patients with insufficient collateral circulation and 43 patients with well-developed collateral circulation were divided into two groups. In addition, the Thrombolysis in Myocardial Infarction (TIMI) flow grade** **was used to angiographically evaluate coronary perfusion after PCI. Flow grade in coronary arteries is classified as grade 0 (no flow), grade 1 (penetration without perfusion), grade 2 (partial perfusion), or grade 3 (complete perfusion) [10].

Venous blood samples for routine clinical chemistry and blood cell count analyses were collected in clot activators and EDTA (ethylenediaminetetraacetic acid) tubes, respectively. All analyses without adropin were performed at presentation. Routine clinical chemistry parameters (creatinine, total cholesterol, triglycerides, high-density lipoprotein, low-density lipoprotein) were obtained on routine chemistry analyzers from Roche (Cobas; Roche Diagnostics, Basel, Switzerland). Blood cells count parameters (leukocytes, lymphocytes, platelets, granulocytes, and hemoglobin) were derived from a complete blood count (CBC) measured on a BC-6800 (Mindray, Shenzhen, China ). For the adropin test, the samples were taken within the first 24 hours of admission in tubes with clot activator and gel and were centrifuged at 3500 rpm for 10 minutes and the serum samples obtained were portioned and stored at -80°C. Absorbance measurements for serum adropin levels using a commercial ELISA (enzyme-linked immunosorbent assay) kit (Shanghai Korain Biotech, Shanghai, China) were performed on a microplate reader (AccuReader, Metertech Inc., Taipei, Taiwan).

Statistical analysis

For descriptive statistics, mean ± standard deviation was used to give continuous data with normal distribution. A median with minimum-maximum values was applied for continuous variables without normal distribution. Numbers and percentages were used for categorical variables. The Shapiro-Wilk, Kolmogorov-Smirnov, and Anderson-Darling tests analyzed the normal distribution of the numerical variables.

The Independent Samples t-test compared two independent groups where numerical variables had a normal distribution. For the variables without normal distribution, the Mann-Whitney U test was applied in comparing two independent groups. The Pearson Chi-Square and Fisher's Exact tests were used to compare the differences between categorical variables in 2x2 tables. The Fisher Freeman Haltontest was used in RxC tables.

The receiver operating characteristic (ROC) analysis using the DeLong method with the Youden index was used to determine the optimum serum adropin cut-off value that predicts the development of insufficient CCC. The area under the characteristic (AUC) curve and the corresponding 95% confidence interval (CI) were calculated. 

Based on the appropriate cut-off value of the serum adropin level, specificity, sensitivity, positive, and negative predictive values, positive, and negative likelihood ratios were also calculated for the parameters with AUC value.

For statistical analysis, Jamovi (Version 2.2.5.0, The Jamovi Project, www.jamovi.org) and JASP (Version 0.16.1, https://jasp-stats.org/) were used. The significance level (p-value) was determined at 0.05 in all statistical analyses.

## Results

There were 84 patients in the study. We randomized 41 (48.8%) patients with insufficient CCC and 43 (51.2%) patients with well-developed CCC. The demographic and clinical characteristics were similar in the groups (p>0.05). We detected significant differences between the groups in white blood cell and neutrophil counts and serum adropin levels. The patients with insufficient CCC had significantly higher white blood cell (12.3 ± 3.7 vs. 10.4 ± 2.9, p=0.009) and neutrophil (9.0 ± 3.8 vs. 7.1 ± 2.4, p=0.004) counts than those with well-developed CCC. The serum adropin levels in patients with insufficient CCC were significantly lower than in those with well-developed CCC (196.3 [131.5 - 837.0] pg/mL vs. 235.5 [171.9 - 1124.2] pg/mL, p<0.001) (Table 1).

**Table 1:** Demographic and clinical characteristics and laboratory investigations of the study groups ^†^mean ± standard deviation, ^‡^n (%), ^§^median [min-max], *Pearson Chi-Square or Fisher's Exact test, **Independent Samples T-Test, ***Mann-Whitney U test, CCC: coronary collateral circulation

			Patients with				
	Overall (n=84)		Insufficient CCC (n=41)		Well-developed CCC (n=43)		p-value
Age (year) ^†^	59.1 ± 11.9		59.4 ± 11.8		58.8 ± 12.1		0.805**
Sex ^‡^							
Male	62 (73.8)		32 (78.0)		30 (69.8)		0.385*
Female	22 (26.2)		9 (22.0)		13 (32.5)		
Smoking ^‡^	27 (32.1)		13 (31.7)		14 (32.5)		0.999*
Diabetes mellitus ^‡^	20 (23.8)		12 (29.2)		8 (18.6)		0.447*
Hypertension ^‡^	44 (52.4)		27 (65.8)		17 (39.5)		0.058*
Hemoglobin (g/dL) ^†^		14.5 ± 1.9		14.5 ± 1.5		0.956*	
White blood cell count (mm^3^) ^†^		12.3 ± 3.7		10.4 ± 2.9		0.009*	
Neutrophil count (mm^3^) ^†^		9.0 ± 3.8		7.1 ± 2.4		0.004*	
Lymphocyte count (mm^3^) ^§^		1.8 [0.6 – 7.3]		2.1 [0.7 – 7.3]		0.856***	
Platelet count (mm^3^) ^†^		247.2 ± 73.7		252.8 ± 59.0		0.690*	
Creatinine (mg/dL) ^§^		0.9 [0.6 – 1.4]		0.9 [0.7 – 1.7]		0.281***	
Total cholesterol (mg/dL) ^†^		193.7 ± 40.1		188.8 ± 46.1		0.590*	
Triglyceride (mg/dL) ^§^		159.0 [55.0 – 474.0]		140.0 [39.0 – 852.0]		0.695***	
High-density lipoprotein (mg/dL) ^†^		40.4 ± 9.9		38.3 ± 10.6		0.353*	
Low-density lipoprotein (mg/dL) ^†^		121.6 ± 36.3		115.1 ± 36.4		0.402*	
Adropin (pg/mL) ^§^		196.3 [131.5 – 837.0]		235.5 [171.9 – 1124.2]		<0.001***	

The angiographic and interventional treatment characteristics of coronary artery disease (CAD) are given in Table 2. The right coronary artery was the most frequently target vessel (60.7%) among all patients. The majority of the patients presented with STEMI (69 %). The TIMI thrombus grade and the outcomes of PCI are detailed in Table 2. 

**Table 2:** Comparison of the groups regarding angiographic and interventional treatment characteristics of coronary artery disease ^‡^n (%), ^§^median [min-max], *Pearson Chi-Square, Fisher's Exact or Fisher Freeman Halton test, **Mann-Whitney U test MI: myocardial infarction, TIMI: Thrombolysis in Myocardial Infarction, PCI: percutaneous coronary intervention, CCC: coronary collateral circulation

		Patients with		
	Overall (n=84)	Insufficient CCC (n=41)	Well-developed CCC (n=43)	p-value
Target vessel ^‡^				
Right coronary artery	51 (60.7)	19 (46.3)	32 (74.4)	0.012*
Left descending artery	26 (30.9)	16 (39.0)	10 (23.2)	
Circumflex artery	7 (8.3)	6 (14.6)	1 (2.3)	
Type of myocardial infarction ^§^				
ST Elevation Myocardial Infarction (STEMI)	58 (69.0)	24 (58.5)	34 (79.0)	0.071*
Non-ST Elevation Myocardial Infarction (NSTEMI)	26 (30.9)	17 (41.5)	9 (20.9)	
Interval between last severe chest pain and intervention(hr) ^§^	4.0 [1.0 – 10.0]	3.0 [2.0 – 6.0]	4.0 [1.0 – 10.0]	0.042**
TIMI thrombus grades ^‡^				
1	3 (3.6)	2 (4.8)	1 (2.3)	0.749*
2	2 (3.3)	1 (2.4)	1 (2.3)	
3	79 (94.0)	38 (92.6)	41 (95.3)	
Outcome of PCI ^‡^				
Complete recanalization (TIMI 3)	76 (90.4)	37 90.2)	39 (90.6)	0.999*
Slow-flow/no-reflow (TIMI ≤2)	8 (9.5)	4 (9.7)	4 (9.3)	

There were significant differences in the frequencies of the target vessel and the median interval between the last severe chest pain and intervention. The right coronary artery was the more frequently detected target vessel in patients with well-developed CCC (74.4% vs. 46.3%), and the circumflex artery was more frequent in patients with insufficient CCC (14.6% vs. 2.3%) (p=0.012). The median interval between the last severe chest pain and the intervention was significantly higher in patients with well-developed CCC than those with insufficient CCC (p=0.042).

TIMI thrombus grade 3 was detected in 94.0% of the patients. Successful PCI was obtained in 90.4% of the patients. The comparison of the other characteristics revealed no significant difference between the groups (p>0.05). 

**Figure 1:**
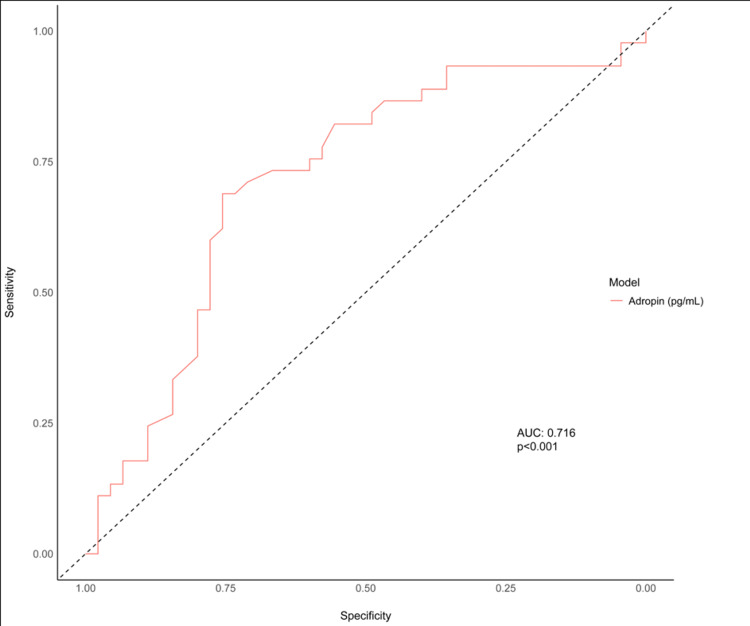
The Receiver Operating Characteristics (ROC) curve analysis of serum adropin in predicting insufficient CCC (AUC: area under curve). ROC: receiver operating characteristics, AUC: area under curve, CCC: coronary collateral circulation

The Receiver Operating Characteristics (ROC) curve analysis revealed that the cut-off value of serum adropin higher than 211 pg/mL had sensitivity and specificity values of 68.9% and 75.6%, respectively, in predicting poor coronary collateral vessel formation (AUC=0.716, CI 95%: 0.611-0.806, p<0.001) (Figure 1).

Logistic regression analysis revealed that the circumflex and left descending coronary artery as the target vessel, NSTEMI as the type of myocardial infarction, and serum adropin level were the independent risk factors for the prediction of insufficient coronary collateral vessel formation (p<0.05) (Table 3).

**Table 3:** Logistic regression analysis of the risk factors in predicting insufficient CCC OR: Odds ratio, CI: confidence interval, NSTEMI: Non-ST elevation myocardial infarction, STEMI: ST elevation myocardial infarction

	Crude OR (95%CI)	Crude p-value	Adjusted OR (95%CI)	Adjusted p-value
Target vessel: ref.=right coronary				
Left descending coronary	2.7 (1.06 – 6.87)	0.037	3.74 (1.21 – 11.59)	0.022
Circumflex coronary	11.55 (1.32 – 100.92)	0.027	40.84 (2.63 – 634.1)	0.008
Type of myocardial infarction: NSTEMI vs. STEMI	2.56 (1.02 – 6.41)	0.045	3.41 (1.07 – 10.88)	0.038
Adropin level	0.99 (0.99 – 1)	0.006	0.99 (0.99 – 1)	0.022
White blood cell count	1.19 (1.04 – 1.36)	0.012	1.04 (0.78 – 1.4)	0.766
Neutrophil count	1.23 (1.06 – 1.43)	0.007	1.22 (0.89 – 1.69)	0.222

## Discussion

In this study, we found that there is a relationship between adropin levels and CCC in patients with acute myocardial infarction. The serum adropin levels in patients with insufficient CCC were significantly lower than in those with well-developed CCC. In addition, there were significant differences in the frequencies of the target vessel and the median interval between the last severe chest pain and intervention. The right coronary artery was the more frequently detected target vessel in patients with well-developed CCC. Logistic regression analysis revealed that the circumflex artery as the target vessel, NSTEMI as the type of myocardial infarction, and serum adropin level were the independent risk factors for the prediction of insufficient CCC.

In the presence of severe coronary stenosis or occlusion, CCC helps maintain myocardium vitality by providing alternative blood flow to the ischemic myocardium. CCC has beneficial effects on infarct size, ventricular remodeling and functions, and mortality in patients with myocardial infarction [10-11]. The CCC are normally collapsed and dysfunctional. Coronary collateral vessels become functional when ischemic events develop. The development of CCC is thought to occur in two stages; angiogenesis and arteriogenesis. Angiogenesis is the development of new vessels by sprouting and invagination from a pre-existing plexus [12]. Severe stenosis of a coronary artery causes a decrease in post-stenotic pressure, and redistribution of blood to the ischemic area occurs by dilation of the vessels developed by angiogenesis. This is called arteriogenesis [13].

The development of CCC is promoted by various factors: hypoxia, hypoperfusion, shear stress, cytokines, and clinical factors such as time of occlusion [14], diabetes mellitus, severity of CAD vessels and coronary ectasia [15].

Adropin is a protein hormone secreted from the liver, which is important for maintaining energy homeostasis and insulin sensitivity [3]. Aydin et al. showed that adropin was expressed in the brain, cerebellum, kidney, heart, liver, pancreas and vascular tissues of diabetic rats [16]. In previous studies, an ischemia model was created in animal experiments and it was observed that adropin treatment increased perfusion and capillary density [5].

Adropin plays an important protective role in cardiovascular endothelial functions as increasing the level of endothelial nitric oxide synthetase (eNOS) by activating vascular endothelial growth factor receptor 2 (VEGFR2) -phosphatidylinositol-3-phosphate kinase pathway activation (P13K-Akt) and VEGFR2 extracellular signal-regulated kinase (ERK1/2) pathways [17]. We claim that the relationship between adropin and CCC is the result of adropin stimulating eNOS [18]. Another result of our study was that NSTEMI as a type of myocardial infarction was the independent risk factor for the prediction of insufficient CCC. We think that this result is due to the higher probability of complete occlusion and more severe stenosis in patients with STEMI. Moreover, the positive correlation between well-developed CCC and increased interval between severe chest pain and intervention is not surprising. Since closed and non-functional collateral arteries need time to become functional, it is expected to be more frequent as this period increases.

This study had several limitations. The number of patients included in this study was limited; larger study groups are needed to confirm the relationship between adropin and CCC. Since the follow-up time is not long in our study, we do not know the effects of high adropin levels on clinical long-term outcomes. Furthermore, the development of CCC is a multifactorial process. We do not have data on many factors such as physical activity, presence of angina before infarction, genetic factors, etc.

## Conclusions

In our study, we found that CCC secondary to ischemia as a result of coronary artery occlusion was associated with increased serum adropin levels. Larger studies are needed to investigate the effects of adropin on CCC. If this relationship can be demonstrated in larger studies, perhaps future studies on the therapeutic use of adropin to enhance the development of CCC can be planned.
